# Depression, Gaming Disorder, and Internet Addiction in Adolescents with Autism Spectrum Disorder

**DOI:** 10.3390/bs15040423

**Published:** 2025-03-26

**Authors:** Masaru Tateno, Yukie Tateno, Tomohiro Shirasaka, Kotaro Nanba, Eri Shiraishi, Ryotaro Shimomura, Takahiro A. Kato

**Affiliations:** 1 Tokiwa Child Development Center, Tokiwa Hospital, Tokiwa 3-1-6-1, Minami-ku, Sapporo 0050853, Japan; yukimasata@yahoo.co.jp (Y.T.); urotauko1985@yahoo.co.jp (K.N.); erieri-o@f3.dion.ne.jp (E.S.); rshimomura1119@gmail.com (R.S.); 2 Child Mental Health Clinic, Department of Neuropsychiatry, Sapporo Medical University Hospital, S-1 W-16, Chuo-ku, Sapporo 0608543, Japan; 3 Department of Psychiatry, Teine Keijinkai Medical Center, Maeda 1-12-1-40, Teine-ku, Sapporo 0060811, Japan; shirasaka.t@gmail.com; 4 Department of Neuropsychiatry, Graduate School of Medical Sciences, Kyushu University, Maidashi 3-1-1, Higashi-ku, Fukuoka 8128582, Japan; kato.takahiro.015@m.kyushu-u.ac.jp

**Keywords:** autism spectrum disorder, neurodevelopmental disorder, depression, behavioral addiction, gaming disorder, internet gaming disorder, internet addiction

## Abstract

Adolescents with autism spectrum disorder (ASD) often have various psychiatric comorbidities, particularly depression. In recent years, gaming disorder (GD) and Internet addiction (IA) have been identified as common comorbidities of ASD. We administered three self-administered screening instruments to adolescents with ASD to assess the severity of depression, GD, and IA. The participants were 10–18-year-olds with ASD. They were asked to complete three questionnaires to assess depressive symptoms, GD, and IA: the Patient Health Questionnaire for Adolescents (PHQ-A), Ten-Item Internet Gaming Disorder Test (IGDT-10), and Internet Addiction Test (IAT). The total IGDT-10 score was calculated in two different ways: the original scoring version (IGDT-10-OV) and the modified version (IGDT-10-MV). Of the 74 respondents, 24.3% had moderate or severe depressive symptoms, 8.1% were identified as having possible GD according to the IGDT-10-OV, 39.2% were identified as having possible GD according to the IGDT-10-MV, and 27.0% were positive for IA according to the IAT. Two-group comparisons revealed that depressive symptoms were more strongly associated with IA than with GD. IA was associated with more depressive symptoms than GD. Since adolescents with ASD have difficulties with social communication, they are prone to isolation and feelings of loneliness. Longer screen times due to social isolation may be a risk factor for the development of GD/IA. Adolescents with ASD often exhibit a preference for visual processing but may struggle with verbal communication. Thus, they may find online spaces more comfortable for them to alleviate their feelings of loneliness.

## 1. Introduction

The Internet has become an indispensable tool in our daily lives. A large-scale survey conducted by the Japanese government reported an Internet usage rate of 98–99% among 10–50-year-olds (Cabinet Office, 2024). However, in recent years, various problems related to excessive Internet use have attracted significant social interest. Internet addiction (IA) is defined as an addiction to all sorts of activities on the Internet, including gaming and social media usage (Griffiths, 1996; Shaw & Black, 2008; Young, 1998). As the number of Internet users increases, problems related to Internet use become more serious, particularly among adolescents (Ko et al., 2005; Lam, 2014). Several cultural and social factors in Japan may contribute to the development of IA among adolescents. Japan is a highly digitalized society, where smartphones, gaming consoles, and high-speed Internet are easily accessible. The widespread availability of technology makes it easier for adolescents to engage in prolonged gaming and Internet use. The lack of strict national regulations on gaming time and Internet use for minors may contribute to the development of IA in Japan.

It is well established that IA is more serious among those who use the Internet for online gaming (Mihara & Higuchi, 2017). Gaming is one of the most popular leisure activities among adolescents, particularly among boys. Online games are commonly played in modern times. Previous studies have demonstrated that teenagers who play online gaming spend increasingly longer hours online (Higuchi et al., 2021). According to a 2023 survey by the Japanese Government, more than 60% of adolescents aged 12–18 played online gaming, with approximately 30% of them spending more than 3 h per day on weekdays (Children and Families Agency, 2024).

The American Psychiatric Association proposed preliminary diagnostic criteria for Internet gaming disorder (IGD) in the *Fifth Edition of the Diagnostic and Statistical Manual of Mental Disorders (DSM-5)* in 2013 (APA, 2013). Although the diagnostic criteria were included in Section III as one of the “Conditions for Further Study”, this declaration eliminated the differences in the concepts of pathological gaming and provided a common understanding among researchers. Consequently, several reliable and reproducible studies have been conducted on this topic.

Furthermore, in 2019, the World Health Organization adopted the 11th revision of the *International Classification of Diseases (ICD-11)*, identifying excessive gaming as a psychiatric disorder (WHO, 2019). Gaming disorder (GD) is characterized by a pattern of persistent or recurrent gaming behavior, manifested by impaired control over gaming, increasing priority given to gaming, to the extent that gaming takes precedence over other life interests and daily activities, and the continuation or escalation of gaming despite the occurrence of negative consequences. This pattern of gaming behavior results in marked distress or significant impairment in important areas of functioning. The classification of GD as a psychiatric disorder in ICD-11 has raised public awareness, prompting more parents to seek medical advice for children with excessive gaming habits.

Excessive Internet use is known to cause a variety of physical and psychological problems among people of all ages (Achab et al., 2011; Ko et al., 2012; Mannikko et al., 2015; Mihara & Higuchi, 2017; Tateno et al., 2022). IA and GD are associated with multiple psychiatric conditions, with depression being the most common one (Dalbudak et al., 2013; Evren et al., 2019; Liu et al., 2018; Ostinelli et al., 2021). Sleep problems are known to have a significant impact on children’s health. High-quality sleep is crucial for children’s development during adolescence. However, GD and IA are common among adolescents, and sleep problems are often reported as the first symptom of these conditions. In a survey of pediatricians and general psychiatrists who serve as primary care providers for children’s mental health issues in Japan, the results demonstrated that consultations related to GD are already common for them (Tateno et al., 2025). As for the clinical symptoms of patients with GD at the time of consultation, both pediatricians and general psychiatrists frequently encounter sleep problems in these cases. Additionally, the survey revealed that pediatricians often deal with somatic symptoms, whereas psychiatrists primarily address behavioral problems and psychiatric symptoms such as anxiety. A large-scale survey of 15,786 Japanese students aged 12 to 18 demonstrated that adolescents with short sleep duration may be at a higher risk of experiencing psychotic symptoms (Morishima et al., 2020). Another study conducted in Japan reported that prolonged Internet use may be linked to disrupted sleep patterns, leading to decreased academic performance and reduced physical activity among adolescents (Kawabe et al., 2016).

Currently, depression is known to be the most prevalent psychiatric condition in adolescents, although it was previously considered a psychiatric disorder that primarily affects adults. Nevertheless, variations in survey timing and methodology make it difficult for us to compare the results of studies on depression directly. Additionally, in Japan, studies focusing on depression among adolescents are extremely limited.

The school refusal rate among junior high school students in Japan has been reported to be 6.7% (MEXT, 2024), indicating that depression is not uncommon in this age group (Finning et al., 2019). In this study, we focused on adolescents with ASD who sought medical care. However, since establishing a control group was not feasible, we referred to the results of a school-based survey that was conducted using the same evaluation method. A survey of 7765 adolescents in Aomori Prefecture revealed that 13.6% of the respondents exhibited moderate or more severe depressive symptoms (Adachi et al., 2020). Additionally, 3.4% of the respondents had “moderately severe” depressive symptoms, while 1.5% had “severe” depressive symptoms. Notably, many adolescents experience depressive symptoms.

Another psychiatric disorder that is a frequent comorbidity of IA and GD is autism spectrum disorder (ASD) (Murray et al., 2022; Simonelli et al., 2024; So et al., 2017). In the DSM-5, ASD is defined as a neurodevelopmental disorder alongside attention deficit/hyperactivity disorder (AD/HD) (APA, 2013). ASD is characterized by persistent deficits in social communication and social interaction across multiple contexts and restricted, repetitive patterns of behavior (RRB), interests, or activities. Recent results from large-scale surveys in Japan have reported a prevalence of 3.2% among 5-year-olds in Hirosaki city, Aomori, and 3.1% among 32-month-olds in Hamamatsu city, Shizuoka (Nishimura et al., 2019; Saito et al., 2020). A report by the Autism and Developmental Disabilities Monitoring (ADDM) Network, in the U.S., also reported a prevalence of ASD of 2.8% (Maenner et al., 2023). Based on these reports, the prevalence of ASD should be considered to be about 3%.

Systematic reviews have been conducted to explore the link between GD, IA, and depression. These reviews consistently report a significant association between both GD and IA and depression (Cerniglia et al., 2017; Ostinelli et al., 2021). However, findings on the relationship between GD, IA, and ASD have been inconsistent across studies (Kervin et al., 2021; Murray et al., 2022; Shane-Simpson et al., 2016). Therefore, in this study, we focused solely on adolescents with ASD and examined the relationships between GD, IA, and depression.

This study investigated the degree of depressive symptoms, GD, and IA in adolescents with ASD. Adolescents with ASD are prone to longer Internet use because they are more likely to be alone due to an impairment of social skills. Extended Internet usage could be related to a higher risk of developing IA. However, there are suggestions that prolonged Internet use in individuals with ASD is not due to addiction but rather a consequence of their social impairments, which are one of the characteristics of ASD. Because they spend less time with others, their screen time naturally increases. Therefore, we considered it beneficial to examine whether prolonged Internet use in individuals with ASD constitutes IA or is merely longer engagement time.

It should be noted that in this paper, for simplicity and consistency, we use the term “GD” as an inclusive concept that encompasses IGD in the DSM-5, because GD in ICD-11 and IGD are very similar.

## 2. Materials and Methods

### 2.1. Subjects

Study participants were recruited from the outpatient clinic of Tokiwa Child Development Center, Tokiwa Hospital, Sapporo, Japan. All eligible adolescents with a confirmed diagnosis of ASD in the age range of 10–18 years, except those with moderate-to-severe intellectual disabilities or serious psychiatric symptoms, were invited to participate in the study. The clinical diagnosis of ASD was confirmed by consensus among a multi-professional team, including board-certified child psychiatrists, as per the DSM-5. The inclusion criteria were adolescents with ASD who played online games for at least 1 h per week. The child and adolescent psychiatrist in charge asked potential participants if they had played games for more than an hour per week, and if they answered yes, they were informed about the study’s aims and methods. The attending physician obtained informed assent and informed consent from the participants and their guardians, respectively, prior to enrollment in the study. Participants were requested to complete the questionnaire.

### 2.2. Study Questionnaire

The study questionnaire consisted of questions about sociodemographic characteristics (including age and sex), gaming behavior (duration of gaming on weekdays and weekends), and the three data collection tools described below. The time required to complete the questionnaire was typically between 5 and 10 min.

### 2.3. Self-Administered Screening Instruments

#### 2.3.1. The Patient Health Questionnaire for Adolescents (PHQ-A)

The PHQ-A is a self-administered questionnaire adapted from the Patient Health Questionnaire-9, which is one of the most popular screening tools for depression in adults (Gilbody et al., 2007; Kroenke et al., 2001). The PHQ-A was designed to identify symptoms of major depression during the past 2 weeks in adolescents aged 12–18 years (Johnson et al., 2002). The PHQ-A consists of nine questions based on the nine items of DSM diagnostic criteria for major depression. Each item was scored on a 4-point Likert scale: 0 = not at all; 1 = several days; 2 = more than half the days; and 3 = nearly every day. The total scores on the PHQ-A ranged from 0 to 27. In general, the severity of depressive symptoms was assessed based on the following total scores: 0–4, minimal or no depression; 5–9, mild depression; 10–14, moderate depression; 15–19, moderately severe depression; and 20–27, severe depression. In this study, we determined the cutoff score to be 15 points to define the depression group.

#### 2.3.2. The Ten-Item Internet Gaming Disorder Test (IGDT-10)

The IGDT-10, a self-rating scale, was developed by Király et al. (2017). The IGDT-10 was designed to screen for IGD according to the diagnostic criteria described in Section III of the DSM-5 (APA, 2013). The IGDT-10 consists of ten items regarding the frequency of gaming-related problems during the previous 12 months on a 3-point Likert scale: 0 = never; 1 = sometimes; and 2 = often. IGDT-10 has ten items, whereas the diagnostic criteria for IGD in the DSM-5 have only nine items, as the IGDT-10 assesses functional impairment via two questions. The total IGDT-10 score ranged from 0 to 9. The original version of the IGDT-10 determined the cutoff to be five points.

#### 2.3.3. Internet Addiction Test (IAT)

Young developed the IAT as a diagnostic instrument for IA based on the DSM-IV criteria for pathological gambling (Young, 1998). The IAT consists of 20 questions regarding the frequency of Internet use. All questions begin with a sentence such as “How often do you…?.” For example, a question asks “How often do you find that you stay online longer than you intended? (Q1).” The response choices range from 1 = rarely to 5 = always. The original cutoff point for IA is ≥70, although several studies recommend a reasonable cutoff value of ≥50 (Dufour et al., 2016; Tateno et al., 2018). A Canadian research group demonstrated that a cutoff of 50 could be appropriate for screening subjects with probable IA among 3938 healthy adolescents (Dufour et al., 2016); hence, we applied this cutoff in this study. The reliability and validity of the Japanese version of the IAT have also been investigated (C. M. Lai et al., 2015; Mak et al., 2014; Tateno et al., 2023).

### 2.4. Two Scoring Methods of IGDT-10

We used two IGDT-10 scoring methods: the original version (OV) and the modified version (MV).

The original cutoff points proposed by the developer of the IGDT-10 comprise five or more responses of 2 = often (original version) (Király et al., 2017). That is, it is considered positive only when “often” is selected. As mentioned earlier, although the IGDT-10 consists of 10 questions, the maximum score is 9 points, because it assesses dysfunction in daily life using two questions, Q9 and Q10. In this study, the original scoring method was defined as the IGDT-10 OV.

In 2022, a research group at Kurihama Medical and Addiction Center (KMAC) proposed a less stringent scoring method, using 1 = sometimes applicable, and a total score of ≥5 was treated as testing positive on the IGDT-10 (Mihara et al., 2022). The results of the KMAC study (n = 5096, aged 10–29 years) demonstrated that the sensitivity and specificity were sufficiently high, and a receiver operating characteristic analysis revealed that the proposed modified scoring method had a higher screening performance than the original scoring method. The proposed cutoff for the modified scoring for GD was 5 points, which is the same as that of the original scoring. In this study, we refer to this modified scoring method as IGDT-10 MV.

### 2.5. Statistical Analysis

Statistical analyses were performed using StatFlex Ver.7 (Artec Inc., Osaka, Japan). The reliability of the three tools was assessed by calculating their internal consistency (Cronbach’s α coefficient). The Mann–Whitney U test was used to compare the means of two groups, while analysis of variance was used for three or more groups. Pearson’s correlation coefficient was calculated to investigate the association between these two factors. The chi-squared test was used to evaluate whether there were significant differences in categorical variables between the groups. Statistical significance was set at *p* < 0.05.

### 2.6. Ethics

The study protocol was approved by the Ethics Committee of Tokiwa Hospital prior to data collection (TH-200713). The study was conducted in accordance with the principles of the Declaration of Helsinki. Informed assent and informed consent were obtained from the participants and their guardians, respectively.

## 3. Results

The total number of respondents was 86. Of these, eight respondents who had multiple missing data points and four with no gaming time per week were excluded from the statistical analyses. Thus, 74 cases were considered valid. The results are summarized in Table 1.

The internal consistency of the three self-administered scales used in this study was assessed by calculating Cronbach’s α. The results indicated acceptable reliability, with Cronbach’s α values of 0.826 for PHQ-A, 0.865 for IGDT-10, and 0.936 for IAT.

Of the 74 respondents, 24.3% had moderately severe or severe depressive symptoms, 8.1% were identified as having possible IGD according to the IGDT-10-OV, 39.2% were identified as having possible IGD according to the IGDT-10-MV, and 27.0% showed positive results for IA according to the IAT.

When the presence and absence of significant depressive symptoms were compared (Table 2), the group with depressive symptoms spent more time online on both weekdays and weekends. However, there was no significant difference in the gaming times for weekdays and weekends. The depressive group not only had longer Internet usage time but also showed significantly higher IAT scores than the non-depressive group, indicating that they were more severely addicted to the Internet. The average IAT score of the depressive group exceeded the cutoff of 50 points, which has been recommended in some studies (Dufour et al., 2016; Tateno et al., 2018).

Regarding the two-group comparison between the IGD and non-IGD groups (Table 3), the GD group showed significantly longer gaming time on weekdays and weekends, longer Internet usage time on weekdays and weekends, and higher scores on the IAT. In summary, the IGD group was more likely to be addicted to the Internet. However, no significant difference was found between the two groups regarding the severity of depressive symptoms when comparing those with and without IGD.

Similarly, we performed a two-group comparison between the IA and non-IA patients (Table 4). The IA group spent a much longer time gaming on weekdays and weekends, spent a much longer time using the Internet on weekdays and weekends, and had more severe IGD than the non-IA group. In addition, the IA group had significantly more severe depressive symptoms than the non-IA group.

**Figure 1:**
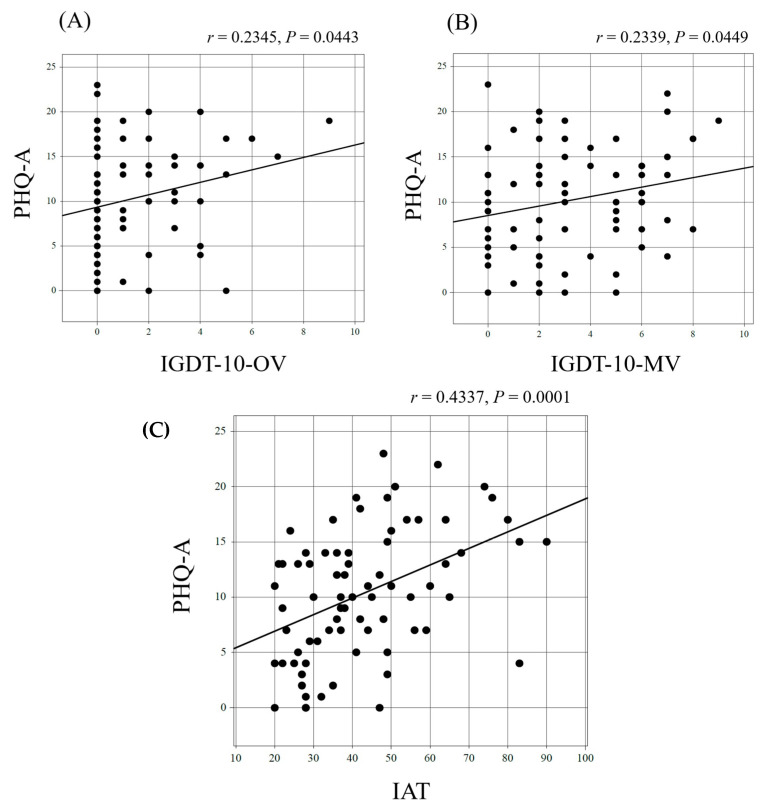
Correlation of the severity of depressive symptoms with gaming disorder and Internet addiction. (**A**) shows the correlation between PHQ-A and IGDT-10-OV, (**B**) shows the correlation between PHQ-A and IGDT-10-MV, and (**C**) shows the correlation between PHQ-A and IAT. PHQ-A: The Patient Health Questionnaire for Adolescents; IGDT-10: the Ten-Item Internet Gaming Disorder Test; OV: original version; MV: modified version; IAT: Internet Addiction Test.

The relationship between GD (Figure 1A,B), IA (Figure 1C), and the severity of depressive symptoms was examined using correlation coefficients for the total scores on both scales. Significant correlations were found for all two-group combinations; however, the correlations were stronger for IA than for GD in relation to depressive symptoms.

## 4. Discussion

The present results revealed that approximately one-fourth of the adolescents with ASD had moderately severe or severe depressive symptoms, as assessed by the self-rating scale PHQ-A. A school survey in Japan demonstrated that 13.6% of adolescents had a moderate degree of depressive symptoms, and 4.9% had moderately severe or severe depressive symptoms (Adachi et al., 2020). These results suggest that adolescents with autism have a higher risk of depression than adolescents with a typical developmental history. The differences in survey timing and methodology make it difficult to directly compare the results of studies on depression. However, since reliable studies on the prevalence of depression among adolescents in Japan are extremely limited, we referred to the results of school surveys.

It has been reported that females of all ages have a higher prevalence of depression than males. Depressive symptoms tend to become more severe as the age of an individual increases from early adolescence to adulthood. Similar trends were also observed in our study participants with ASD. A two-group comparison of the presence and absence of depression demonstrated that the mean age of the depressive group was significantly higher than that of the non-depressive group.

Patients with ASD often have various and multiple comorbidities (Bougeard et al., 2021; M. C. Lai et al., 2014; Mutluer et al., 2022). The presence of depressive symptoms in adolescents with autism requires careful assessment of the contributing factors to the depressive symptoms. The present results suggest that excessive gaming and Internet use may be involved in the development of depression in adolescents with ASD.

Adolescents with ASD and depressive symptoms spent significantly more time on the Internet on both weekdays and weekends than those without depressive symptoms. A study involving 3938 Canadian high school students using the IAT to assess IA with cutoff points of 50, consistent with the present study, reported that 18% were considered to have IA (Dufour et al., 2016). In our study, 27% of participants were screened as having possible IA, indicating that the risk of IA must be high in individuals with ASD.

The results of this study showed no significant difference in daily gaming time, suggesting that adolescents with ASD would engage in online activities other than online gaming for a longer time in comparison with time spent on online gaming. It has been reported that individuals with ASD prefer written communication to verbal communication and prefer watching the screen due to their predominance of visual information processing (Gillberg, 2021; Volkmar et al., 2014). In recent years, video games have often been played together by multiple players online, but individuals with ASD tend to play in isolation because of social impairment, resulting in frequent solo games (Dell’Osso et al., 2023; Orsmond & Kuo, 2011). When game players enjoy video games with multiple players, such as the Massively Multiplayer Online Role-Playing Game (MMORPG), it is common to play while chatting via voice chat tools; however, adolescents with ASD may not be good at online multiplayer games, because they are not good at verbal communication and simultaneous processing that requires immediate responses.

A nationwide survey of 5096 randomly selected 10–29-year-olds in Japan reported an IGDT-10-MV positivity rate of 11.3% (Mihara et al., 2022). In the present study, the IGDT-10-MV positivity rate was very high at 39.2%. This may be because the present study included only those who reported playing games habitually and only teenagers who were prone to develop problematic gaming behaviors. Overall, individuals with ASD are thought to be more prone to having GD.

In adolescents with ASD, IA was strongly associated with depressive symptoms but weakly associated with GD. A comparison of the two groups with and without GD showed no statistically significant differences in the severity of depressive symptoms. However, regarding IA, the severity of depressive symptoms was significantly higher in the IA group than in the non-IA group. Although the DSM-5 diagnostic criteria for IGD include the item “relive negative moods,” game players with ASD might be more likely to play games for mood modification. While a few of them could have GD, many others might play the game for fun in an appropriate manner.

The two core features of ASD in both the DSM-5 and ICD-11 are deficits in social communication and social interaction and RRB (APA, 2013; WHO, 2019). Regarding the association between ASD and GD/IA, there are two hypotheses: one is that patients with ASD spend more time alone due to social impairment, resulting in spending more time playing games or using the Internet, and the other is that they are preoccupied with gaming because of their obsessive behavior as a symptom of RRB. In the clinical experience of the first author, several adolescents with ASD have been playing an old model offline video game console as an RRB for many years. When an adolescent with ASD plays games as an RRB, they may also have an obsession with the time of ending the game. Consequently, a diagnosis of GD would not be appropriate in such cases, as the individual exhibits no impaired control over gaming.

Hikikomori is a form of severe social withdrawal characterized by physical isolation at home (Kato et al., 2019, 2020). Hikikomori must meet the following criteria: severe social isolation at home, continuous social isolation for at least 6 months, and significant functional impairment associated with social isolation. Approximately one-third of hikikomori cases are diagnosed with ASD or other neurodevelopmental disorders (Kondo et al., 2013). Patients with ASD who are reclusive by remaining in their homes owing to difficulties in social interaction may spend a lot of time playing games. However, in such cases, even though they play games for a long time to pass the time while they are socially withdrawing, they cannot be diagnosed with GD, because they do not meet the diagnostic criteria for GD.

This study has some limitations. This was a single-site, cross-sectional study, which suggests a lower level of representativeness. Additionally, the sample size was not sufficiently large to draw any conclusions. It was impossible to include a control group. Although sufficient and high-quality sleep is essential for the physical and mental health of adolescents, it was not assessed in this study. Depressive symptoms were evaluated solely through self-reported questionnaires. We were unable to assess any psychiatric comorbidities other than depressive symptoms, GD, and IA. Most results were based on self-administered questionnaires.

## 5. Conclusions

Adolescents with ASD experience various mental health issues, with depression being one of the most common psychiatric comorbidities in this patient population. The results of the present study suggest that IA, more so than GD, is associated with depressive symptoms in adolescents with ASD. In clinical settings, careful assessment of adolescents with ASD is needed to identify early signs of GD/IA and prevent the development of depression.

## Figures and Tables

**Table 1:** Sociodemographics of the subjects and summary of the screening tools.

Age	14.1 ± 2.4
Sex (M:F)	47:27
Length of gaming (hr/d)	
Weekdays	2.7 ± 2.8
Weekends	3.8 ± 3.5
Length of Internet use (hr/d)	
Weekdays	4.6 ± 3.3
Weekends	6.4 ± 4.0
PHQ-A	10.3 ± 5.9
Depression (≥15)	n = 18 (24.3%)
IGDT-10-OV	1.4 ± 2.0
Screened positive (≥5)	n = 6 (8.1%)
IGDT-10-MV	3.4 ± 2.6
Screened positive (≥5)	n = 29 (39.2%)
IAT	42.6 ± 16.9
Possible IA (≥50)	n = 20 (27.0%)

The resuls are expressed as the mean ± standard deviation. PHQ-A: Patient Health Questionnaire-Adolescent. IGDT-10: The Ten-Item Internet Gaming Disorder Test. IAT: Internet Addiction Test. OV: original version, MV: modified version, IA: internet addiction.

**Table 2:** Two-group comparison between presence and absence of depressive symptoms.

	Depressive (n = 18)	Non-Depressive (n = 56)	*p* Value
Age	15.0 ± 2.0 *	13.8 ± 2.4	* *p* = 0.0384
Sex (M:F)	8:10	39:17	*p* = 0.0533
Length of gaming (hr/d)			
Weekdays	3.6 ± 3.6	2.4 ± 2.4	*p* = 0.2100
Weekends	4.9 ± 4.4	3.4 ± 3.1	*p* = 0.1698
Length of Internet use (hr/d)			
Weekdays	6.1 ± 3.6 *	4.2 ± 3.1	* *p* = 0.0422
Weekends	8.3 ± 4.0 *	5.8 ± 3.8	* *p* = 0.0219
IGDT-10-OV	2.2 ± 2.8	1.1 ± 1.5	*p* = 0.1101
IGDT-10-MV	4.3 ± 3.0	3.1 ± 2.5	*p* = 0.1099
IAT	57.2 ± 17.8 *	37.9 ± 13.8	* *p* = 0.0001

The results are expressed as the mean ± standard deviation. An asterisk (*) denotes a statistically significant difference. IGDT-10: The Ten-Item Internet Gaming Disorder Test. IAT: Internet Addiction Test. OV: original version, MV: modified version.

**Table 3:** Two-group comparison between presence and absence of Internet gaming disorder.

	IGD (+) (n = 29)	IGD (−) (n = 45)	*p* Value
Age	14.1 ± 2.3	14.1 ± 2.4	*p* = 0.8996
Sex (M:F)	22:7	25:20	*p* = 0.0765
Length of gaming (hr/d)			
Weekdays	4.3 ± 3.1 *	1.6 ± 2.0	* *p* = 0.0001
Weekends	6.0 ± 3.6 *	2.4 ± 2.7	* *p* < 0.0001
Length of Internet use (hr/d)			
Weekdays	5.7 ± 3.2 *	4.0 ± 3.2	* *p* = 0.0329
Weekends	7.7 ± 3.9 *	5.5 ± 3.9	* *p* = 0.0199
PHQ-A	11.4 ± 5.3	9.6 ± 6.1	*p* = 0.1771
IAT	52.1 ± 18.9 *	36.4 ± 12.2	* *p* = 0.0002

The results are expressed as the mean ± standard deviation. An asterisk (*) denotes a statistically significant difference. IGD: Internet Gaming Disorder. PHQ-A: Patient Health Questionnaire-Adolescent. IAT: Internet Addiction Test.

**Table 4:** Two-group comparison between presence and absence of Internet addiction.

	IA (+) (n = 20)	IA (−) (n = 54)	*p* Value
Age	14.4 ± 2.1	14.0 ± 2.5	*p* = 0.4701
Sex (M:F)	12:8	35:19	*p* = 0.7024
Length of gaming (hr/d)			
Weekdays	4.9 ± 3.3 *	1.9 ± 2.1	* *p* = 0.0002
Weekends	7.0 ± 3.5 *	3.8 ± 2.8	* *p* = 0.0001
Length of Internet use (hr/d)			
Weekdays	7.0 ± 3.5 *	3.8 ± 2.8	* *p* = 0.0005
Weekends	9.3 ± 3.8 *	5.3 ± 3.5	* *p* = 0.0014
PHQ-A	14.1 ± 4.9 *	8.9 ± 5.6	* *p* = 0.0002
IGDT-10-OV	3.1 ± 2.6 *	0.7 ± 1.2	* *p* = 0.0003
IGDT-10-MV	5.3 ± 2.6 *	2.7 ± 2.2	* *p* = 0.0002

The results are expressed as the mean ± standard deviation. An asterisk (*) denotes a statistically significant difference. IA: Internet Addiction. PHQ-A: Patient Health Questionnaire-Adolescent. IGDT-10: The Ten-Item Internet Gaming Disorder Test. OV: original version, MV: modified version.

## Data Availability

The data that support the findings of this study are available from the corresponding author (M.T.) upon reasonable request. Requests will be considered on an individual basis based on ethical considerations and study requirements.
